# The Clinic Movement: A New Medico-Psychological Institution

**Published:** 1914-01-03

**Authors:** Edwin L. Ash

**Affiliations:** Director of the London Nerve Clinic and School of Psychotherapy


### THE CLINIC MOVEMENT.

### A New Medico-Psychological Institution.

By EDWIN L. ASH, M.D., Lond., Director of the London Nerve Clinic and School of Psychotherapy.

CONSIDERABLE interest attaches to the evident present-day movement for the establishment of institutions providing special treatments for those who come between the very poor and the well-to-do—a movement that concerns a comparatively new branch of administrative medicine in this country. For this is a matter that not only concerns a very large class of persons whose circumstances have prevented them obtaining help in various directions either from the experienced consultant or from a voluntary charity, but it is of importance to the modern practitioner. The modern practitioner who wishes to keep abreast of the latest advances desires opportunities of seeing for himself what claim various new methods have upon his serious attention.

The hardship of the industrious man who has to maintain a good social position and support a large family on a small income in regard to special treatments is great, for he fears to lose caste by going to a hospital whilst the consulting rooms of the West End bid fair to cripple him when the conduct of an obstinate chronic illness of wife, children, or self is in question. The same applies to the needy widow left to keep up a good position on a hopelessly inadequate income, as well as to the single-worker of either sex whose wage does not suffice more than to allow of a comfortable living in a very moderate way. Thus to secure one of its main objects the ideal clinic should not be a charitable institution; but, whilst affording assistance to the very poor, should be self-supporting. Small payments should be made by those who can afford them, arranged so far as possible on a scale graduated according to their means; and fees should be charged for post-graduate lectures and classes.

It is clearly to the advantage of the general public that clinics should be established, because not only do they thus afford special opportunities of educating professional workers in respect of treatment not always provided by the hospitals, but it is safe to say that no therapeutic method that is unworthy can long survive the keen criticism to which its practice is submitted in the full light of a clinical institution. In the future the development of the clinic will probably include accommodation for resident patients derived from those classes who can afford reasonable payments for board and treatment so as to supply the necessary funds for the maintenance of the in-patient department. One of London's most pressing needs in this direction has been the establishment of clinical institutions for patients suffering from nervous and mental disorders. Till recently there was practically no accommodation in the Metropolitan general hospitals for systematic treatment of such persons; but during the last two or three years departments for mental disorders in charge of experts have been established in at least two of the leading hospitals in London—namely, St. Mary's and University College; although it does not appear that any arrangement for the systematic treatment of patients has been made at either.

At the present day it would be absurd to say that there is no such thing as systematic treatment for mental disorders, as medical psychology has advanced sufficiently to be able to indicate several successful lines on which disordered nerves and weakened mind can be treated. The London Nerve Clinic, recently opened at 71 Baker Street, W., offers advantages of the kind in question to persons of moderate means suffering from mental and nervous ailments, being also organised as a school of psycho-therapy. It is a lamentable thing that at the present time there is no such thing as a systematic course of instruction at any of the medical schools in connection with the important part played by mental and spiritual influences in the production and arrest of such disorders, and the new institution will be the first of its kind to offer such facilities under medical supervision. The new clinic will provide opportunities for doctors, students, and nurses to attend lectures and demonstrations on the practice of curative suggestion (with and without hypnosis), as well as combined psycho-electrical methods. Although opened quite recently, a number of patients are in attendance on most afternoons each week; whilst already a following of professional workers interested in the important subject of medical psychology has been attracted to the clinic. The first lecture was given on Wednesday, November 26, by the Director, who took for his subject the question of "Hypnoidal States" as an aid to the use of suggestion in treatment. Various modern investigators have found restful states of the mind, such as those which immediately precede natural sleep, are extremely useful from this point of view and have certain definite advantages over true hypnotic states in many cases.

A lecture was also given on December 10 by Dr. Francis G. Scott, assistant in charge of the routine work of the clinic, who demonstrated the induction of hypnosis to an audience of medical men, pointing out the difficulties to be met with, as well as the precautions to be taken. Great interest is invariably taken in demonstrations of hypnotic phenomena, but little scientific advantage is to be gained from repeated experiments of this kind, and it has been decided that the deeper stages of hypnosis, with the striking phenomena of somnambulism, will be demonstrated to medical men on occasion, but will not form a necessary part of the general course of instruction.

Amongst other objects of the institution will be the placing of medical psychology on a sounder basis, both in the eyes of the medical profession and of the public. It will also act as a publishing centre for the issue of books and pamphlets promoting its object. At present the clinic is open for the reception of patients from 3.30 until 6.0 on Tuesday, Wednesday, Thursday, and Saturday afternoons, but the hours of attendance will be extended in accordance with the requirements of the developing work. Further lectures are being arranged, and during the coming term special attention will be given to the requirements of nurses, for whose benefit a course is being mapped out. It is to be noted that this clinic is independent of any other medico-psychological institution or psycho-therapeutic association, and being privately supported is not appealing for funds.

